# Determinants of bacteriophage Qβ survival under ionizing radiation: roles of hydration, mineral matrices, and viral features

**DOI:** 10.3389/fmicb.2026.1958185

**Published:** 2026-10-08

**Authors:** Alicia Rodríguez-Moreno, Kristina Beblo-Vranesevic, Ester Lázaro

**Affiliations:** 1 Centro de Astrobiología (CAB), CSIC-INTA, Torrejón de Ardoz, Madrid, Spain; 2 Escuela de Doctorado, Centro Estudios Postgrado, Universidad Autónoma de Madrid, Cantoblanco, Spain; 3 Applied Aerospace Biology Department, Institute of Aerospace Medicine, German Aerospace Center (DLR), Cologne, Germany

**Keywords:** bacteriophage Qβ, ionizing radiation, regolith simulants, space microbiology, viral radiotolerance

## Abstract

**Introduction:**

Understanding how viruses persist under extreme physicochemical conditions is essential for both extreme microbiology and astrobiology. While the effects of a great variety of individual stressors have been explored, comparative analyses across different environmental and viral determinants remain limited. In particular, the respective contributions of radiation, hydration state, mineral matrices, and intrinsic viral properties to viral persistence remain largely unexplored.

**Methods:**

We investigated the response of bacteriophage Qβ to increasing doses of ionizing radiation under different physicochemical conditions. Viral survival was quantified in liquid and desiccated systems, in the presence of Martian (MGS-1) and Lunar (LMS-1) regolith simulants, and at different initial viral concentrations. In addition, the susceptibility to ionizing radiation of a thermotolerant mutant was also evaluated. Dose-response relationships were analyzed using log-logistic or log-linear models depending on data structure.

**Results:**

Analyses of the effect of initial viral concentration produced model-dependent results, indicating that any concentration-dependent effect on the radiation response should be interpreted with caution. In liquid medium, MGS-1 (12.5% w/v) modified the response of Qβ to ionizing radiation under the experimental conditions tested, producing a shift in the dose–response relationship and altering inactivation kinetics. However, at a fixed radiation dose of 750 Gy, increased survival was observed only at the highest MGS-1 concentration tested (100% w/v), whereas LMS-1 did not show evidence of a comparable effect under the same experimental conditions. Desiccation dramatically enhanced viral radiotolerance, increasing survival by several orders of magnitude. Finally, the thermotolerant mutant exhibited substantially higher survival under ionizing radiation, indicating that properties of the virion contribute to stress tolerance.

**Discussion:**

Our results demonstrate that viral resistance to ionizing radiation depends on both intrinsic viral features and environmental factors. Among these, hydration state appears as the dominant determinant of survival, while the presence of mineral matrices may exert different effects. This integrative framework highlights the need to consider multiple variables when evaluating viral persistence in extreme environments.

## 1 Introduction

The study of the remarkable resilience of life across a wide range of extreme environments has been of great help to define the boundaries of habitability, a question highly relevant to astrobiology (Horneck et al., 2010). While cellular extremophiles have been extensively investigated as models for life under extraterrestrial conditions (Beblo et al., 2009, 2011; Kish et al., 2009; Garcia-Descalzo et al., 2020; Beblo-Vranesevic et al., 2022; Bourmancé et al., 2025; Arribas Tiemblo et al., 2026a) far less is known about how viruses respond to similar stressors. Understanding viral persistence under extreme physicochemical conditions is increasingly relevant, both for assessing the limits of life and for informing planetary protection strategies. Within this framework, most studies have traditionally focused on the survival and dispersal of cellular microorganisms, particularly bacteria and bacterial spores (Nicholson et al., 2005), because of their potential to contaminate extraterrestrial environments. However, viruses are intrinsically linked to microbial communities and may influence their persistence, evolution, and ecological dynamics. Consequently, understanding viral survival under particular space-relevant conditions is also important for assessing the risks associated with forward contamination.

Viruses are ubiquitous across all known environments, including those considered extreme (Säwström et al., 2008; Yoshida-Takashima et al., 2012; Munson-McGee et al., 2020; Gil et al., 2021). The most abundant viruses are bacteriophages, or phages, which outnumber their bacterial hosts by an order of magnitude (Hendrix et al., 1999; Suttle, 2005). In their extracellular form, viruses exist as virions, which are inert structures that, in many cases, are more resistant than cellular organisms to environmental challenges. In addition, viruses, particularly those with an RNA genome, are characterized by high mutation rates and short replication times (Holland et al., 1982; Duffy et al., 2008; Sanjuán et al., 2010; Domingo et al., 2021). These features lead to the generation of extremely large and genetically heterogeneous populations (Seoane et al., 2026) with a strong capacity for rapid adaptation (Domingo et al., 2012; Andino and Domingo, 2015), as demonstrated in multiple laboratory experiments under controlled selective pressures (Lázaro et al., 2003; Elena, 2016; Geoghegan and Holmes, 2018; Castiglioni et al., 2024; Singhal et al., 2024; Turner and Chao, 2024; Andrews et al., 2026). Altogether, this combination of traits makes viruses powerful yet underexplored model systems for investigating the persistence of biological structures under extreme conditions, including those found in space or extraterrestrial environments (Griffin, 2013; Berliner et al., 2018; de la Higuera and Lázaro, 2022).

Among the environmental stressors relevant to astrobiology, ionizing radiation represents one of the most restrictive factors for biological survival (Leuko and Rettberg, 2017; Moeller et al., 2017; Koschnitzki et al., 2021). High-energy radiation, including X-rays and gamma rays, can damage biomolecules both directly—through ionization and molecular disruption leading to protein oxidation, DNA strand breaks, and base modifications—and indirectly through the generation of reactive oxygen species (ROS) produced by water radiolysis (Riley, 1994; Azzam et al., 2012). These indirect effects are often dominant in aqueous systems, where water acts as both a mediator and amplifier of radiation-induced damage. On planetary surfaces lacking protective magnetic fields or atmospheres with an ozone layer, such as Mars, organisms are exposed to significantly higher radiation fluxes than on Earth (Dartnell et al., 2007; Matthiä et al., 2016), making radiation tolerance a key determinant of survival.

In this context, environmental factors such as mineral matrices and hydration state can critically modulate radiation effects. Planetary regolith, the layer of loose material covering solid rock (Mckay et al., 1991), may provide physical shielding or alter the local chemical environment of embedded biological particles. Martian regolith simulants such as MGS-1 (Mars Global Simulant), and lunar analogs such as LMS-1 (Lunar Mare Simulant), have been widely used to study these interactions under controlled laboratory conditions (Cannon et al., 2019; Long-Fox et al., 2023). However, the potential role of mineral substrates in modulating the response of viruses to ionizing radiation is an unexplored question. Notably, different mineral compositions may display distinct physicochemical properties, including radical scavenging capacity or surface reactivity, leading to divergent effects on biological stability (Arribas Tiemblo et al., 2026b).

Equally important is the role of water in determining radiation sensitivity. Because a substantial fraction of radiation-induced damage arises indirectly from water radiolysis, the presence or absence of water can profoundly alter the extent of molecular damage. Under desiccated conditions, the generation and diffusion of reactive species are severely limited, often resulting in increased resistance to radiation-induced damage. This phenomenon has been well documented in cellular systems such as *Deinococcus radiodurans*, where desiccation confers enhanced resistance to both radiation and other stressors, including heat (Mattimore and Battista, 1996; Daly, 2009; Bauermeister et al., 2011). However, whether similar mechanisms operate in viruses, which are non-metabolizing entities lacking repair systems, remains largely unexplored.

In this work, we investigate the response of bacteriophage Qβ, a positive-sense single-stranded RNA virus that infects *Escherichia coli*, to ionizing radiation under a range of physicochemical conditions relevant to extraterrestrial environments. Specifically, we assess how viral concentration, hydration state, and the presence of mineral simulants (MGS-1 and LMS-1) modulate viral survival. However, interactions between all factors were not directly tested and other variables such as pressure, temperature fluctuations, or atmospheric composition were not included. Therefore, direct extrapolation of the results to Martian or Lunar environments should be performed with caution.

The structural organization, high mutation rate, and established use in evolutionary studies (Arribas et al., 2014; Somovilla et al., 2019, 2022; Hossain et al., 2020; Laguna-Castro et al., 2023, 2024, 2025; Rodríguez-Moreno et al., 2025) make bacteriophage Qβ particularly suitable for investigating virus persistence and adaptability under extreme selective pressures. Its genome encodes four proteins involved in replication, capsid assembly, host recognition, and lysis. Among them, the A1 protein is a minor structural component generated by translational read-through of the major capsid protein stop codon, resulting in an extended version that protrudes from the icosahedral capsid (Rumnieks and Tars, 2011). Although present at low abundance within the virion, A1 is essential for viral infectivity and plays a key role in the early stages of infection (Hofstetter et al., 1974). Notably, a previously described non-synonymous mutation (A1956G), producing the amino acid substitution K204E in A1, confers increased resistance to extracellular heat shocks (Lázaro et al., 2018). Because A1 is required for infectivity, changes affecting its susceptibility to environmental damage may have a great effect on viral survival. Whether this mutation also confers protection against ionizing radiation remains unknown and is also addressed in the present study.

By integrating environmental and viral determinants, this study provides a more comprehensive framework to understand the factors governing viral persistence under extreme conditions.

## 2 Materials and methods

### 2.1 Virus production and stock preparation

The plasmid pBRT7Qβ, containing a full-length cDNA copy of bacteriophage Qβ cloned into pBR322 (Taniguchi et al., 1978; Barrera et al., 1993), was used to transform *Escherichia coli* DH5-α. This strain supports intracellular expression of the viral genome but is not susceptible to phage infection due to the absence of the F-pilus. The supernatant, containing released phage particles, from an overnight culture of a transformed colony was used to infect *E. coli* Hfr (Hayes, 1953) in semisolid agar. Individual lysis plaques were isolated, and one randomly selected was used to initiate virus amplification. Briefly, 10^6^ plaque-forming units (pfu) were used to infect an *E. coli* Hfr culture under standard laboratory conditions [37 °C, 250 rpm, 2 h in NB medium: 8 g/L Nutrient Broth (Merck) supplemented with 5 g/L NaCl] using a New Brunswick Scientific Innova 42 incubator shaker (Eppendorf, Enfield, CT, USA). Following incubation, cultures were treated with chloroform (1:20 v/v, 28 °C, 15 min, 850 rpm in a thermomixer) to release intracellular virions, and then centrifuged at 16,000 × g. The resulting supernatant, containing viral particles at titers of approximately 10^1^^1^ pfu/mL, was aliquoted and stored at −80 °C. For subsequent experiments, frozen aliquots were thawed and diluted in phage buffer [25 mM Tris-HCl (pH 7.5), 5 mM MgCl_2_, 1 g/L gelatin (Merck)] to the desired viral concentrations. Once thawed, aliquots were stored at 4 °C and used within 15 days. Viral titers were determined by plaque assay following standard procedures.

The plasmid pBRT7Qβ was also used to engineer a single-mutant virus (Qβ_A1956G_) containing the mutation A1956G. The protocol to obtain this mutant has been previously described (Lázaro et al., 2018).

### 2.2 Susceptibility of bacteriophage Qβ to ionizing radiation in liquid medium

Irradiation was performed using a Gulmay RS 225A X-ray source (Gulmay Medical Ltd., Camberley, UK) operating at 200 kV and 15 mA. No additional beam filtration was applied during irradiation. Samples were positioned 21 cm below the source and exposed to a dose rate of 20 ± 5 Gy/min, as measured with a UNIDOS dosimeter (PTW Freiburg GmbH) prior to each irradiation experiment. Dosimetry was performed to calibrate the irradiation setup but not within regolith-containing samples. Samples were irradiated up to a maximum dose of 3 kGy. All experiments were conducted under oxic conditions at room temperature (approximately 20–25 °C). Sample temperature was not actively controlled during irradiation. The X-ray source was equipped with an internal cooling system to ensure stable instrument operation, and no noticeable heating of the samples was observed during the experiments.

To assay Qβ survival in liquid medium, virus dilutions prepared in phage buffer were distributed into 200 μL aliquots in 96-well microplates and exposed to increasing doses of ionizing radiation. Following irradiation, samples were immediately used for plaque assays to determine virus titers.

To assess the effect of a fixed amount (12.5% w/v) of the Martian regolith simulant MGS-1 (Space Resources Technologies, Oviedo, FL, USA) on viral survival during irradiation, virus dilutions prepared in phage buffer (200 μl, ~10^8^ pfu/mL) were distributed in 96-well plates containing in each well 25 mg of simulant. Prior to irradiation, plates were incubated for 10 min at 25 °C and 150 rpm. Samples receiving different radiation doses were removed from the irradiation chamber at the corresponding time points by transferring an aliquot from the upper part of the virus-regolith mixture to a separate microplate. Because regolith particles partially sedimented during the experiment, the transferred aliquots contained the finer fraction that remained suspended, whereas most sedimented material remained in the original well. Following completion of the irradiation run, samples were centrifuged at 16,000 × g for 10 min to remove particulate material, and the supernatants were immediately used in plaque assays.

To evaluate the effect of different concentrations of MGS-1 or LMS-1 (Space Resource Technologies), virus dilutions prepared in phage buffer (200 μL, ~10^9^ pfu/mL) were mixed in 2 mL microcentrifuge tubes with the amounts of regolith necessary to reach the concentrations of 25%, 50%, 75%, and 100% w/v. Mixtures were vortexed and irradiated as described above using a fixed dose of 750 Gy. After irradiation, samples were centrifuged at 16,000 × g for 10 min, and the supernatants were immediately used in plaque assays. In parallel, non-irradiated samples were incubated with the same amounts of regolith simulant.

Prior to use, all regolith simulants were sterilized by autoclaving.

### 2.3 Susceptibility of bacteriophage Qβ to ionizing radiation under desiccation

To evaluate the effect of desiccation on viral tolerance to ionizing radiation, two independent protocols, vacuum desiccation (method 1) and surface desiccation on glass disks (method 2), were used. In the first case, virus dilutions (200 μL, ~10^8^ pfu/mL) were placed in 96-well plates and dried using a vacuum concentrator (Concentrator plus/Vacufuge^®^ plus, Eppendorf, Hamburg, Germany) in desiccator mode (D-AQ) at room temperature without additional heating until apparent desiccation was achieved (~4 h). According to the manufacturer's specifications, the system operates at pressures below 20 mbar. The dried plates were then stored overnight within the desiccator and used the following day. Desiccation on solid surfaces, on glass disks, was performed following protocols previously described (Beblo et al., 2009, 2011; Zaccaria et al., 2024). Aliquots (200 μL, ~10^8^ pfu/mL) were deposited onto sterile 1 cm diameter glass disks and allowed to dry for 24 h at room temperature in a laminar flow cabinet under sterile conditions (relative humidity 30% ± 10%).

Dried samples were irradiated as described above. In the case of vacuum desiccation, viral particles were subsequently recovered by resuspension in 200 μL of phage buffer followed by incubation for 10 min at 25 °C and 150 rpm. In the case of desiccation on glass disks, virus recovery took place by placing each disk in 1 mL of phage buffer and vigorous vortexing. In both cases, virus titers were determined by plaque assay.

### 2.4 Overview of experimental conditions

To facilitate comparison among the different assays, the main experimental conditions used throughout the study are summarized in Table 1.

**Table 1:** Summary of experimental conditions assayed.

Experiment	Sections	Figures	Viral concentration	Radiation dose	Hydration state	Regolith simulant	Regolith concentration
Effect of initial viral concentration	2.2 3.1 4.1	Figure 1	10^8^ or 10^9^ pfu/mL	0–2000 Gy	Hydrated	None	0%
Effect of MGS-1 on dose-response curves	2.2 3.2 4.2	Figure 2	10^8^ pfu/mL	0–3000 Gy	Hydrated	MGS-1	12.5% (w/v)
Effect of MGS-1 concentration	2.2 3.3 4.2	Figure 3	10^9^ pfu/mL	750 Gy	Hydrated	MGS-1	0, 25, 50, 75, 100% (w/v)
Effect of LMS-1 concentration	2.2 3.3 4.2	Figure 4	10^9^ pfu/mL	750 Gy	Hydrated	LMS-1	0, 25, 50, 75, 100% (w/v)
Vacuum desiccation	2.3 3.4 4.3	Figure 5	10^8^ pfu/mL	0–3000 Gy	Desiccated (vacuum drying)	None	0%
Desiccation on glass disks	2.3 3.4 4.3	Figure 6	10^8^ pfu/mL	0–3000 Gy	Desiccated (glass disks)	None	0%
Effect of mutation A1956G	2.2 3.5 4.4	Figure 7	10^8^ pfu/mL	0–3000 Gy	Hydrated	None	0%

### 2.5 Data analysis

Statistical analyses were performed using R (version 4.3.2). Dose–response relationships were modeled using four-parameter log-logistic or log-linear functions. The log-logistic model was used for curves with a sigmoidal profile. It has the general form:


$$\begin{array}{l} y\left(D\right)=d+\frac{a-d}{1+{\left(\frac{D}{ED_{\text{50}}}\right)}^{b}} \end{array}$$ (1)

where *y(D)* represents the logarithm of the viral titer at dose *D, a*, and *d* correspond to the upper and lower asymptotes of the response (maximum and minimum log-transformed titers, respectively), *b* is the slope parameter describing the steepness of the curve, and *ED*_*50*_ is the dose corresponding to the midpoint of the response (median effective dose). Because the model is fitted to log-transformed viral titers, the estimated *ED*_*50*_ represents the radiation dose at which the response is halfway between the upper and lower asymptotes in logarithmic scale.

In cases where the dose–response relationship did not exhibit a clear sigmoidal profile, or where the data did not converge toward a lower asymptote within the range of doses tested, a log-linear model was used:


$$\begin{array}{l} y\left(D\right)=i+mD \end{array}$$ (2)

where *y(D)* represents the log-transformed viral titer at dose *D, i* corresponds to the initial value of the response, and *m* is the slope parameter describing the rate of inactivation.

Differences between treatments were assessed by fitting models with either shared or condition-specific parameters and comparing them using analysis of variance (ANOVA). Pairwise comparisons of model parameters were conducted using the *compParm* function. All tests were two-tailed, and statistical significance was set at *p* < 0.05. In specific cases, two-way ANOVAs followed by *post-hoc* Dunnett's tests were conducted.

All experimental conditions were performed in three independent biological replicates, each corresponding to an independently prepared sample subjected to the specified treatment. Data are presented as mean values ± standard deviations. The detection limit of the plaque assay corresponded to a single lysis plaque obtained when plating 100 μL of undiluted sample, equivalent to approximately 10 pfu/mL in the original suspension. In experiments where very low viral titers were anticipated, 200 μL were plated, resulting in a detection limit of approximately 5 pfu/mL. Viral titers were calculated independently for each biological replicate and subsequently averaged. When no plaques were detected in any replicate, samples were considered to be below the detection limit of the assay. For graphical representation and model fitting in logarithmic scale, one of the three replicates was assigned a value of 1 pfu/mL, resulting in a mean titer of 0.33 pfu/mL. These values should be interpreted as indicating complete or near-complete loss of infectivity rather than as precise quantitative estimates.

In cases where baseline viral titers differed between experimental conditions (i.e., differences at dose *D* = 0), survival rates were additionally calculated to normalize the response relative to the corresponding untreated controls. The survival rate was defined as the proportion of viral particles that retained infectivity after exposure to environmental factors, calculated as:

$$\begin{array}{l} \text{Survival rate}=\frac{N}{N_{0}} \end{array}$$ (3)

where *N* is the number of infectious viral particles after irradiation and *N*_0_ is the corresponding non-irradiated control.

## 3 Results

### 3.1 Initial viral concentration influences the apparent dose-response relationship of bacteriophage Qβ to ionizing radiation in liquid medium

To determine whether the initial viral load influences the response to ionizing irradiation in bacteriophage Qβ, we exposed two viral suspensions (10^8^ and 10^9^ pfu/mL) to increasing doses of radiation in liquid medium until reaching 2 kGy. As expected, viral infectivity and survival rates decreased progressively with increasing radiation dose in both conditions (Figure 1). The curves showed a similar overall shape, although a clear displacement toward higher doses was observed for the higher initial virus concentration.

**Figure 1:**
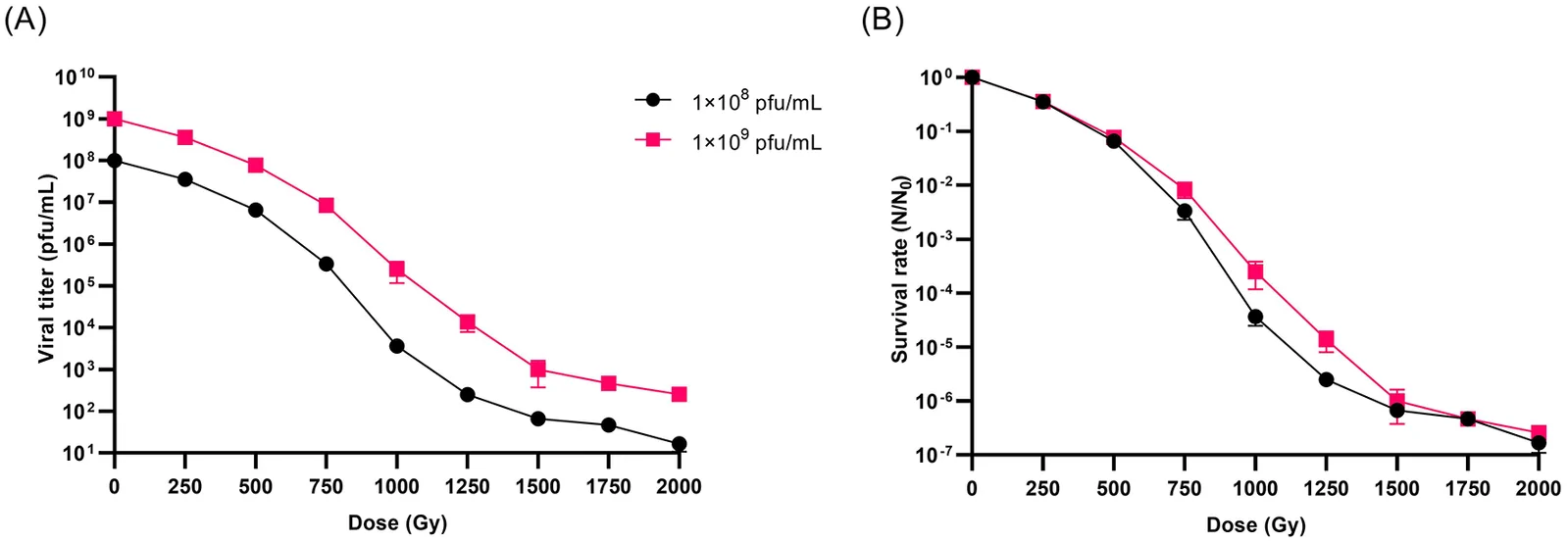
Dose–response relationship of bacteriophage Qβ to ionizing radiation at two initial viral concentrations. **(A)** Viral titers (pfu/mL) as a function of radiation dose for initial concentrations of 10^8^ (black circles) and 10^9^ pfu/mL (pink squares). **(B)** Survival rate of Qβ under the same conditions, calculated relative to the corresponding non-irradiated controls. Data points represent mean values from three independent biological replicates, and error bars indicate standard deviation.

Dose–response relationships were first analyzed using a four-parameter log-logistic model fitted to log-transformed viral titers shown in Figure 1A. Comparison of model fits showed that allowing independent curves significantly improved the fit relative to a single shared model (*p* < 0.001), indicating that the overall dose–response relationships differ between conditions. Consistently, the estimated *ED*_*50*_ values were significantly different (885 ± 17 Gy for the 10^8^ pfu/mL condition and 1,045 ± 32 Gy for the 10^9^ pfu/mL condition; *p* = 0.0005), reflecting a displacement of the fitted dose-response relationship toward higher radiation doses at higher initial viral concentrations. No significant differences were detected in the slope parameter (*b* = 3.47 ± 0.26 for 10^8^ pfu/mL and *b* = 3.09 ± 0.25 for 10^9^ pfu/mL; *p* = 0.287), indicating that no differences in inactivation kinetics were detected under the experimental conditions tested.

To assess whether this effect persisted after accounting for the different initial viral titers, the normalized survival data shown in Figure 1B were analyzed. When fitted using the same log-logistic framework, the same significant differences between *ED*_*50*_ values were again observed, with estimates of 885 ± 20 Gy and 1034 ± 34 Gy for the 10^8^ and 10^9^ pfu/mL populations, respectively. However, analyses performed using normalized data in linear scale yielded no significant differences in either *ED*_*50*_ (*p* = 0.946) or slope (*p* = 0.625). These results indicate that the apparent effect of viral concentration on the dose-response relationship depends on the analytical framework employed and should therefore be interpreted with caution.

### 3.2 Evidence for a protective effect of MGS-1 against ionizing radiation in bacteriophage Qβ

To evaluate the potential radioprotective effect of the Martian regolith simulant MGS-1 on bacteriophage Qβ, dose–response experiments were performed in liquid medium supplemented with 12.5% (w/v) MGS-1. This regolith-to-virus ratio was selected based on previously established protocols (Arribas Tiemblo et al., 2026b). The results showed that the presence of MGS-1 resulted in a modest reduction in viral infectivity (approximately 0.5 log units) during the initial incubation phase prior to irradiation (10 min at 25 °C and 150 rpm; see Section 2.2) and in a clear shift of the dose–response curves toward higher radiation doses, consistent with a protective effect (Figure 2). Previous studies showed that the detrimental effect of MGS-1 on Qβ infectivity in the absence of radiation is largely restricted to the initial stages of exposure (Arribas Tiemblo et al., 2026b). To verify that prolonged contact with the regolith during the irradiation procedure did not contribute substantially to viral inactivation, an independent control experiment was performed under the same conditions but in the absence of radiation. After the initial 10 min incubation with agitation, virus titers decreased from the initial input of 10^8^ pfu/mL to 5.2 × 10^7^ pfu/mL. Then, virus samples were maintained in contact with MGS-1 for an additional 3 h without agitation. Viral infectivity decreased to 2.2 × 10^7^ pfu/mL during the first 15 min and remained stable thereafter for the remainder of the experiment. The magnitude of the initial titer reduction differed slightly from that observed in the dose-response experiment shown in Figure 2, as expected for independent biological assays. Nevertheless, both experiments showed that the negative effect of MGS-1 on infectivity was largely restricted to the initial stages of exposure and did not continue during prolonged incubation.

**Figure 2:**
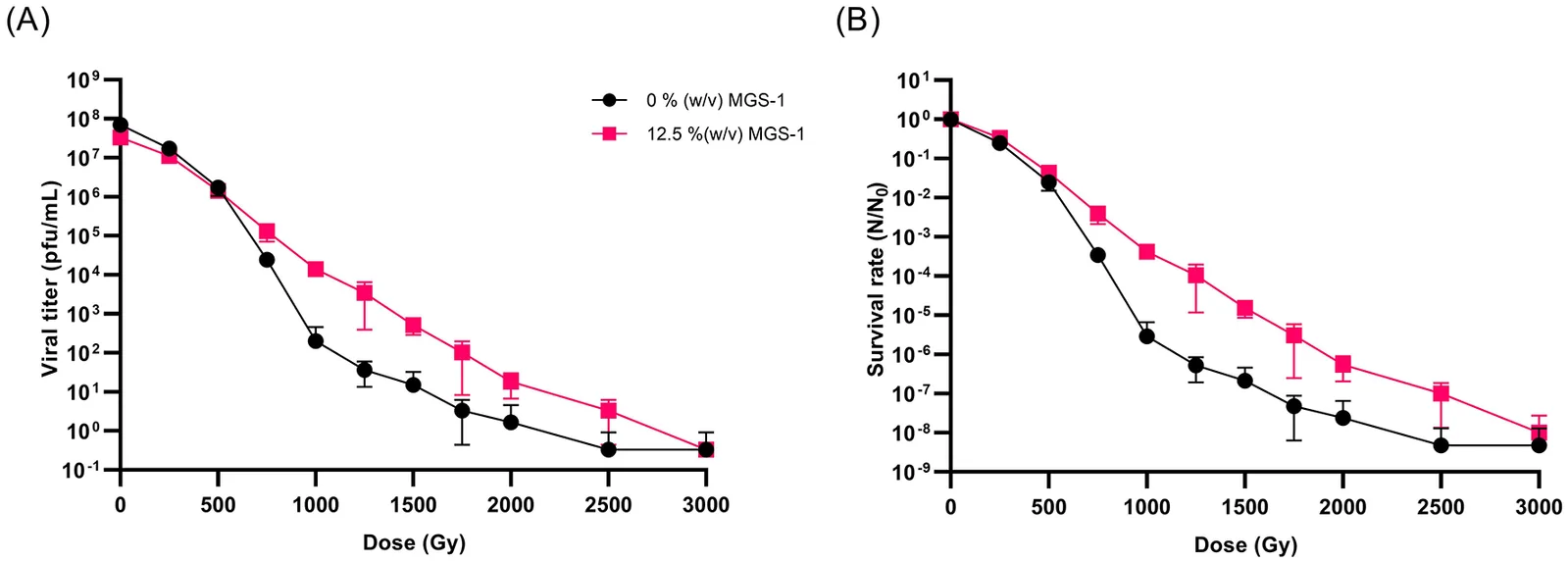
Effect of MGS-1 on the response of bacteriophage Qβ to ionizing radiation in liquid medium. **(A)** Viral titer (pfu/mL) as a function of radiation dose in the absence (0% w/v; black circles) and presence (12.5% w/v; pink squares) of the Martian regolith simulant MGS-1. **(B)** Survival rate under the same conditions, calculated relative to the corresponding non-irradiated controls (*N*_0_ = 7.0 x 10^7^ pfu/ml in the absence of MGS-1 and *N*_0_ = 3.3 x 10^7^ pfu/ml in its presence). Data points represent mean values from three independent biological replicates, and error bars indicate standard deviation.

Dose–response relationships were analyzed using a four-parameter log-logistic model fitted to log-transformed viral titers. Model comparison showed that allowing condition-specific curves significantly improved the fit relative to a shared model (*p* < 0.001), indicating that MGS-1 alters the overall dose–response relationship. The estimated *ED*_*50*_ values were 797 ± 21 Gy in the absence of MGS-1 and 1348 ± 98 Gy in its presence, representing a substantial and significant shift toward higher radiation doses (*p* = 2.07 × 10^−12^). The slope parameter *b* took values of 3.17 ± 0.28 for the control curve in the absence of MGS-1 and 1.90 ± 0.18 for MGS-1-treated samples (*p* = 0.0036), indicating that MGS-1 not only shifts the position of the dose–response curve but also alters its steepness. Together, these results indicate that MGS-1 modifies the response of bacteriophage Qβ to ionizing radiation under the experimental conditions tested.

### 3.3 Differential effects of MGS-1 and LMS-1 on Qβ survival at a fixed radiation dose

Considering the protective effect observed at 12.5% (w/v) MGS-1, we investigated whether increasing the regolith-to-virus ratio further enhances viral survival under ionizing radiation. To this end, viral suspensions were exposed to a fixed radiation dose (750 Gy) while varying the MGS-1 concentration (0, 25, 50, 75, and 100% w/v). As shown in Figure 3A, increasing concentrations of MGS-1 led to a progressive decrease in viral titer in both irradiated and non-irradiated samples, indicating that the presence of regolith negatively affects viral infectivity under basal conditions. Despite this, when survival was expressed relative to the corresponding non-irradiated controls (Figure 3B), a distinct pattern emerged. At the radiation dose tested (750 Gy), survival rates at 25, 50, and 75% (w/v) MGS-1 were similar to, or slightly lower than, those observed in the absence of MGS-1, whereas a marked increase was observed at 100% (w/v). Whether this pattern reflects a threshold response or a dose-dependent interaction between radiation and regolith concentration remains unknown and would require full dose-response analyses across multiple MGS-1 concentrations. In addition, at 100% (w/v), the virus-regolith mixture contained very limited free liquid, and therefore the observed increase in survival may reflect changes in the physicochemical environment of the sample in addition to any specific mineral-mediated effects.

**Figure 3:**
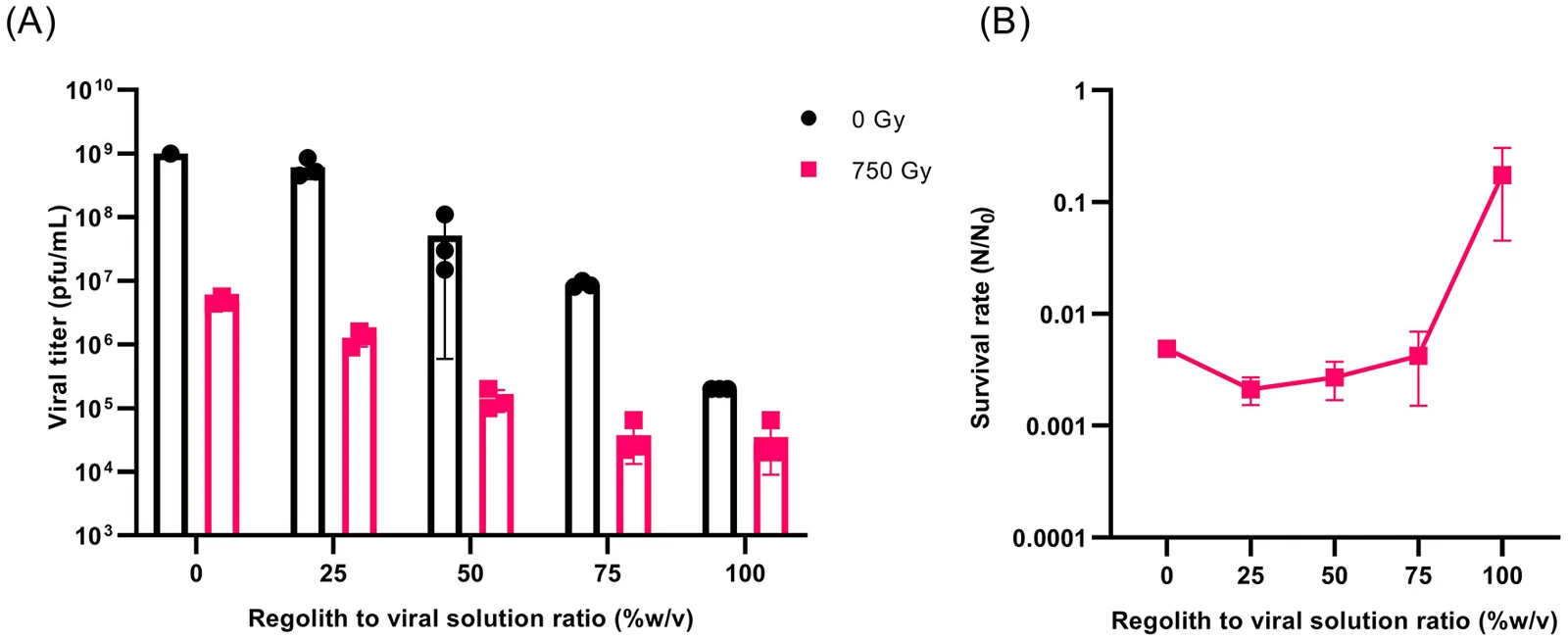
Effect of MGS-1 concentration on the survival of bacteriophage Qβ under ionizing radiation in liquid medium. **(A)** Viral titer (pfu/mL) measured in the absence of radiation (black circles) and after exposure to 750 Gy (pink squares) across increasing MGS-1 concentrations (0–100% w/v). Bars represent mean values, and individual data points correspond to biological replicates. **(B)** Survival rate at 750 Gy as a function of MGS-1 concentration, calculated relative to the corresponding non-irradiated controls. Error bars represent standard deviation from three independent biological replicates.

Statistical analysis using two-way ANOVA revealed significant effects of radiation (*p* < 0.001), MGS-1 concentration (*p* < 0.001), and a significant interaction between both factors (*p* < 0.001), indicating that the impact of radiation at this dose depends on regolith concentration. *Post-hoc* comparisons (Dunnett's test) confirmed that radiation-induced reductions in viral titer were significant across all concentrations.

Direct comparison between Figures 2 and 3 should be made with caution, as both the experimental design and the range of regolith concentrations differ. Figure 2 is based on complete dose–response curves, whereas Figure 3 evaluates survival at a single radiation dose (750 Gy). Because the radioprotective effect of MGS-1 is likely influenced by both regolith concentration and radiation dose, the analysis of a single irradiation dose (750 Gy) provides only a partial view of the response.

To determine whether the lunar regolith simulant LMS-1 exerts a radioprotective effect, viral suspensions were also exposed to a fixed dose of 750 Gy in the presence of increasing concentrations of LMS-1 (Figure 4). In contrast to MGS-1, LMS-1 had only a minor effect on viral infectivity in non-irradiated samples, with viral titers remaining within the same order of magnitude across the concentration range tested (Figure 4A). Upon irradiation, viral titers decreased by approximately three orders of magnitude in all conditions, regardless of LMS-1 concentration. Consistent with these observations, survival rates remained largely unchanged across all LMS-1 concentrations (Figure 4B), showing no evidence of a concentration-dependent protective effect.

**Figure 4:**
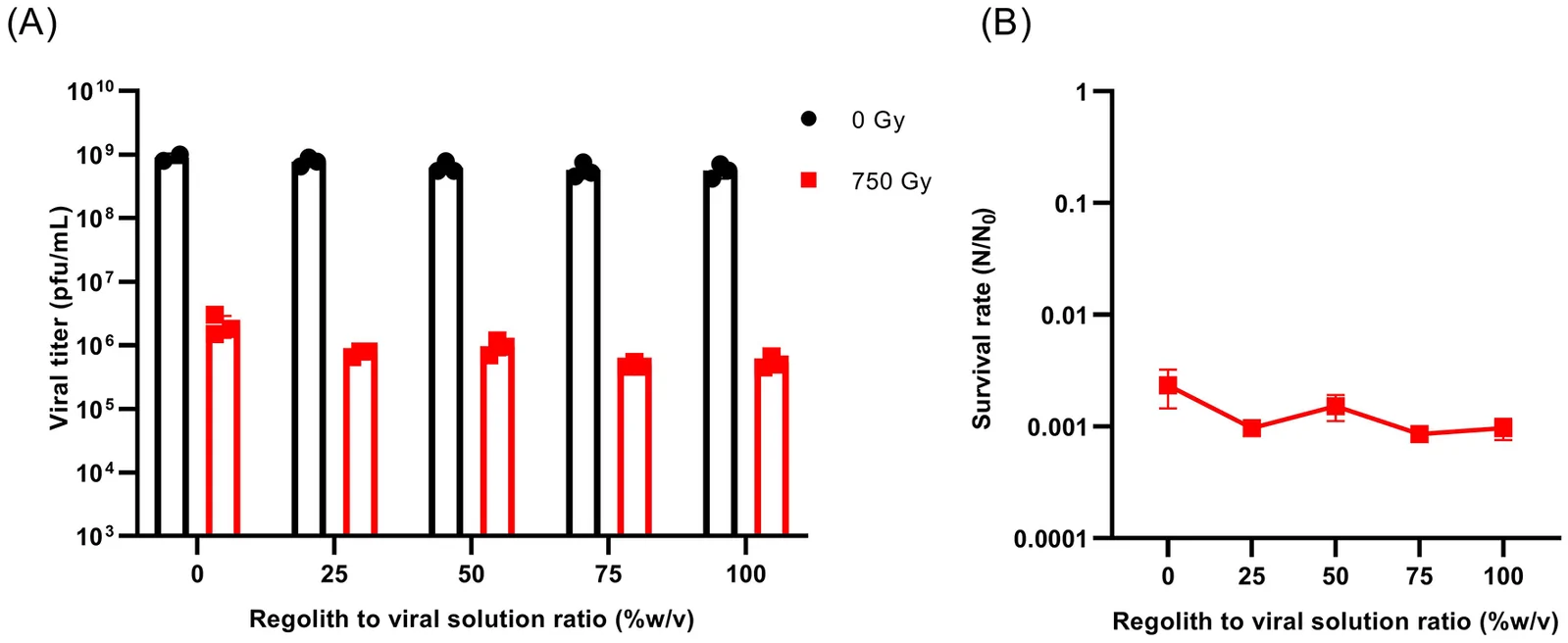
Effect of the lunar regolith simulant LMS-1 on the survival of bacteriophage Qβ under ionizing radiation in liquid medium. **(A)** Viral titer (pfu/mL) measured in the absence of radiation (black circles) and after exposure to 750 Gy (red squares) across increasing LMS-1 concentrations (0–100% w/v). Bars represent mean values, and individual data points correspond to biological replicates. **(B)** Survival rate at 750 Gy as a function of LMS-1 concentration, calculated relative to the corresponding non-irradiated controls. Error bars represent standard deviation from three independent biological replicates.

A two-way ANOVA revealed a significant effect of radiation (*p* < 0.001), accounting for most of the variation in the data, as well as a statistically significant effect of LMS-1 concentration (*p* < 0.001) and a significant interaction between both factors (*p* < 0.01). However, differences in survival rates depending on the presence of LMS-1 were small in magnitude and did not correspond to a consistent increase in survival, indicating that they are unlikely to reflect biologically meaningful radioprotection. *Post-hoc* comparisons using Dunnett's test confirmed that radiation-induced reductions in viral titer were significant at all LMS-1 concentrations tested (all *p* < 0.0001), with no attenuation of the effect relative to irradiated samples in the absence of LMS-1. Taken together, these results indicate that, unlike MGS-1, LMS-1 neither compromises viral infectivity nor provides measurable protection against ionizing radiation under the conditions tested.

### 3.4 Desiccation strongly enhances radiotolerance in bacteriophage Qβ

We investigated the effect of desiccation on the susceptibility of bacteriophage Qβ to ionizing radiation using two independent drying methodologies: vacuum desiccation (method 1) and surface desiccation on glass disks (method 2) (Figures 5 and 6). Both approaches yielded qualitatively similar results, indicating that enhanced radiotolerance was observed under two different desiccation procedures. However, because residual water content was not directly quantified, potential differences in the degree of dehydration achieved by each method cannot be excluded. Desiccation alone caused a reduction in viral infectivity of approximately 1.5–2 log units compared to non-desiccated controls (Figures 5A and 6A). Despite this initial loss, desiccated samples displayed a much more gradual decrease in viral infectivity upon irradiation than non-desiccated ones, which suggests an increase in the resistance to the negative impact of ionizing radiation (Figures 5A and 6A). This difference is even more evident when expressed as survival rates, where desiccated samples retained substantially higher values across the entire dose range (Figures 5B and 6B).

**Figure 5:**
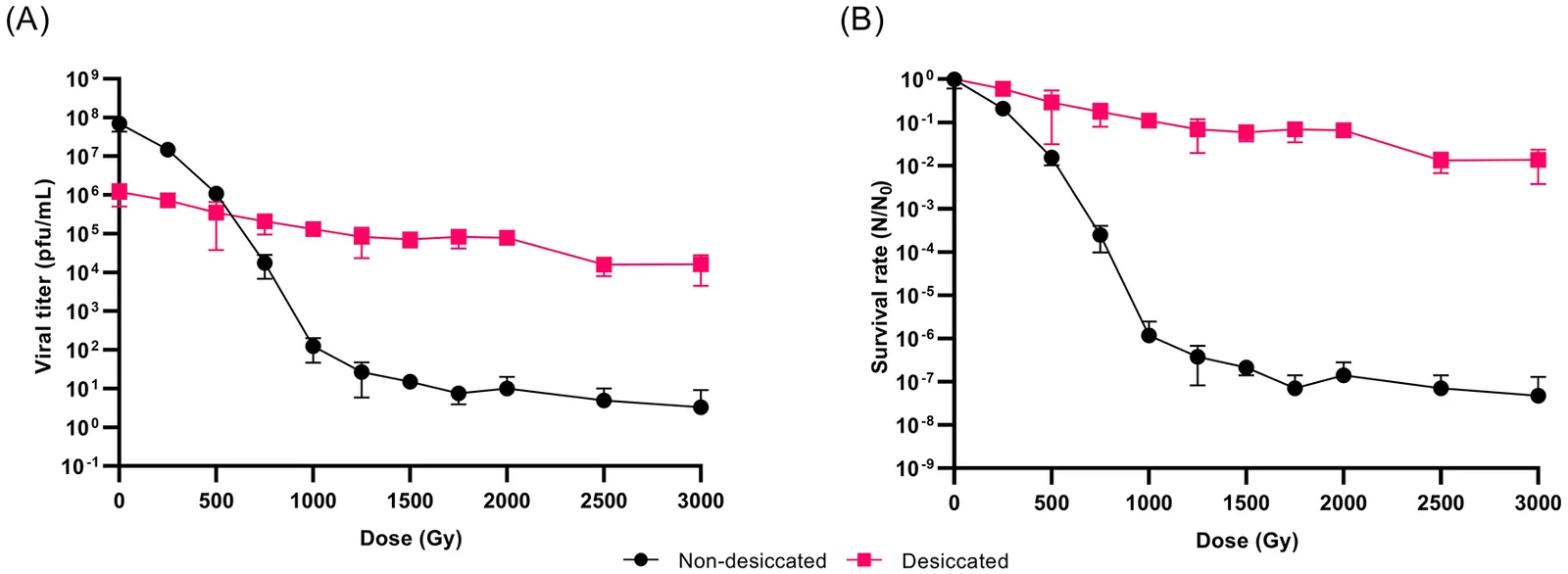
Effect of vacuum desiccation on the response of bacteriophage Qβ to ionizing radiation. **(A)** Viral titer (pfu/mL) as a function of radiation dose for non-desiccated (black circles) and desiccated (pink squares) samples. **(B)** Survival rate under the same conditions, calculated relative to the corresponding non-irradiated controls (*N*_0_ = 7.1 x 10^7^ pfu/ml for non-desiccated samples and *N*_0_ = 1.2 x 10^6^ pfu/ml for desiccated). Data points represent mean values from three independent biological replicates, and error bars indicate standard deviation.

**Figure 6:**
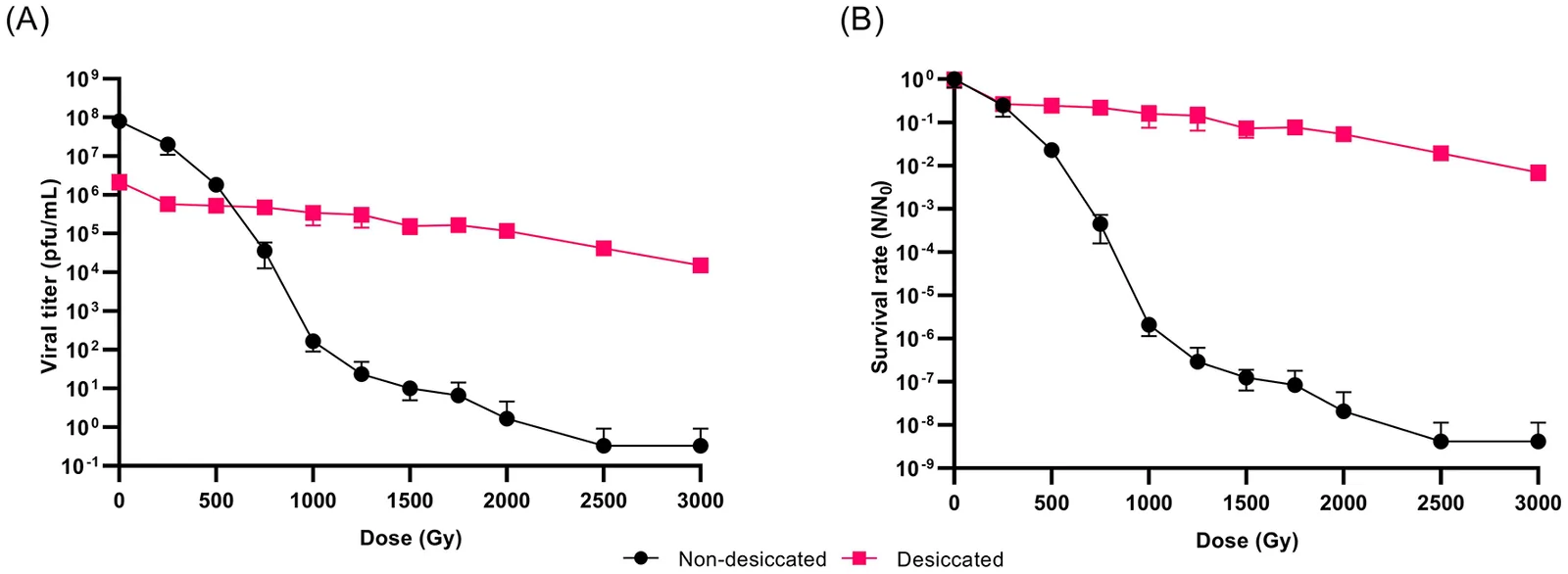
Effect of desiccation on glass disks on the response of bacteriophage Qβ to ionizing radiation. **(A)** Viral titer (pfu/mL) as a function of radiation dose for non-desiccated (black circles) and desiccated (pink squares) samples. **(B)** Survival rate under the same conditions, calculated relative to the corresponding untreated controls (*N*_0_ = 8.0 x 10^7^ pfu/ml for non-desiccated samples and *N*_0_ = 2.2 x 10^6^ pfu/ml for desiccated). Data points represent mean values from three independent biological replicates, and error bars indicate standard deviation.

In both experimental setups, non-desiccated samples exhibited a typical sigmoidal dose–response relationship (*ED*_*50*_ = 739 ± 20 Gy and 803 ± 19 Gy; *b* = 3.39 ± 0.33 and 3.51 ± 0.30 for the data in Figures 5A and 6A respectively), whereas desiccated samples showed a markedly different pattern characterized by an approximately log-linear decline in infectivity across the tested dose range (*m* = −6.05 × 10^−4^ ± 5.06 × 10^−5^ and −6.09 × 10^−4^ ± 3.55 × 10^−5^ for the data shown in Figures 5A and 6A, respectively). The absence of a lower asymptote prevented robust estimation of dose–response parameters such as *ED*_*50*_ for desiccated samples. Notably, the transition from a sigmoidal response in hydrated conditions to an approximately log-linear decay under desiccation suggests a shift in the dominant mechanisms of viral inactivation.

Direct comparison of survival rates at matched radiation doses further illustrates the magnitude of the desiccation effect. Under vacuum desiccation, survival increased from 2.5 × 10^−4^ to 1.8 × 10^−1^ at 750 Gy, and from values close to the detection limit in hydrated samples to 1.4 × 10^−2^ at 3 kGy. Similar results were obtained with desiccation on glass disks (method 2), where survival increased from 4.5 × 10^−4^ to 2.2 × 10^−1^ at 750 Gy and from values below the detection limit to 7.0 × 10^−3^ at 3 kGy. These comparisons indicate that desiccated samples consistently maintained survival rates several orders of magnitude higher than hydrated controls.

### 3.5 A thermotolerance-associated mutation enhances resistance to ionizing radiation

A single mutant containing the non-synonymous mutation A1956G (substitution K204E in the A1 protein), previously shown to increase the resistance to extracellular heat-shocks (Lázaro et al., 2018), was evaluated for its potential role in resistance to ionizing radiation. To investigate whether adaptation to thermal stress correlates with increased radioresistance, we compared the survival of the wild-type virus and the single mutant Qβ_A1956G_ upon exposure to increasing doses of ionizing radiation in liquid medium (Figure 7).

**Figure 7:**
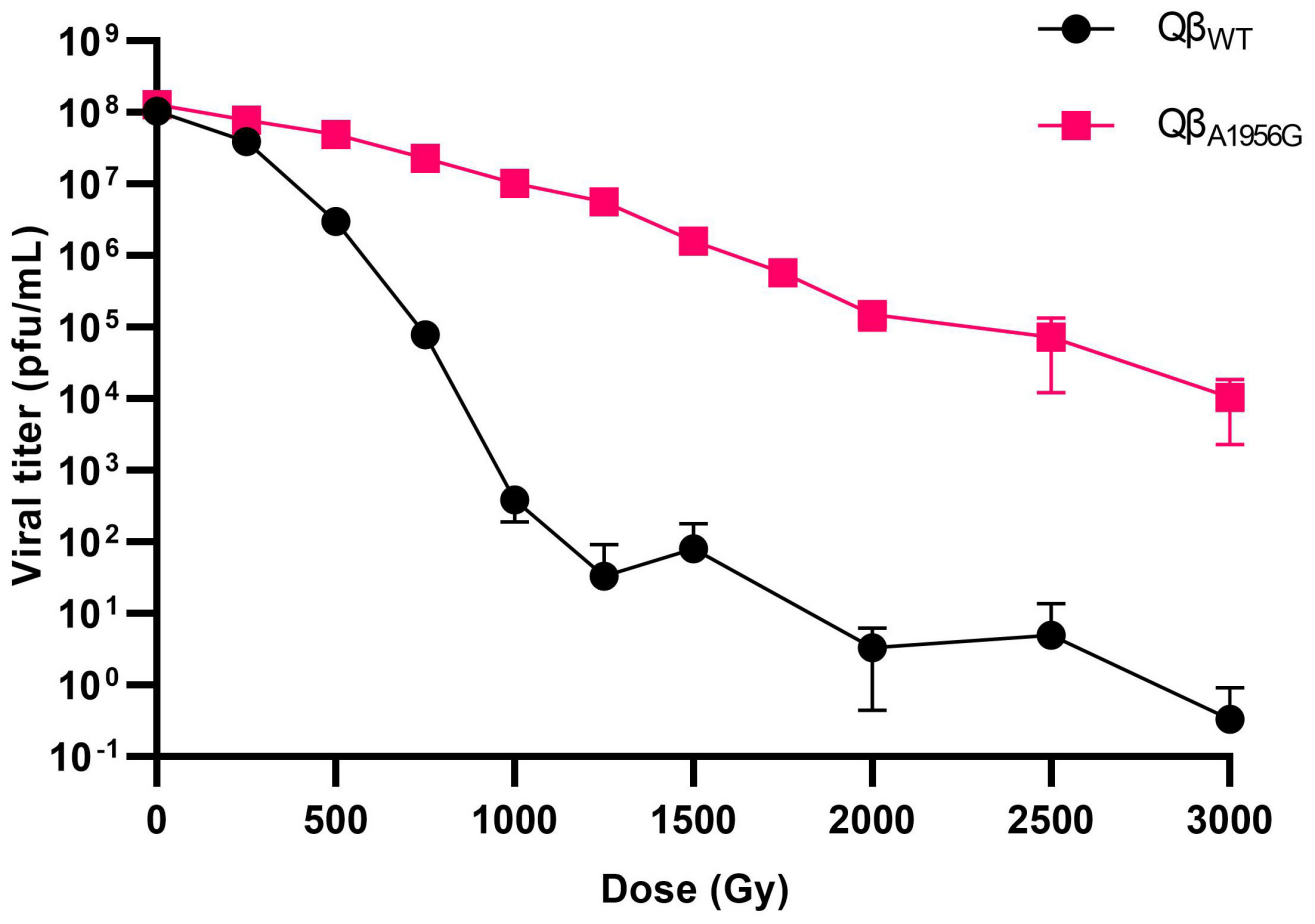
Effect of the A1956G mutation on the response of bacteriophage Qβ to ionizing radiation. Viral titer (pfu/mL) as a function of radiation dose for the wild-type population (Qβ_WT_, black circles) and the A1956G mutant (Qβ_A1956G_, pink squares). Data points represent mean values from three independent biological replicates, and error bars indicate standard deviation.

The wild-type population exhibited a typical sigmoidal dose–response relationship that was well described by a four-parameter log-logistic model. In contrast, the mutant Qβ_A1956G_ displayed a markedly different response, characterized by a gradual decline in viral infectivity across the entire dose range tested and lacking a clearly defined lower asymptote. As a consequence, the response of the mutant was more consistent with an approximately log-linear pattern of inactivation. Because the wild-type and mutant populations were best described by different dose-response models, direct comparison of fitted model parameters was not performed.

In addition to the differences in curve shape, the overall response clearly indicated increased resistance to ionizing radiation in the mutant population. At 750 Gy, infectious titers of the mutant exceeded those of the wild-type population by approximately 2.5 orders of magnitude. The difference became particularly evident at the highest radiation dose (3 kGy), where infectious particles of the mutant remained detectable at titers exceeding 10^4^ pfu/mL, whereas infectivity of the wild-type population was below the detection limit (Figure 7). These results demonstrate that the A1956G mutation, originally selected for increased thermotolerance, also increases survival upon exposure to ionizing radiation, suggesting that adaptation to thermal stress can generate cross-protection against other environmental stressors.

## 4 Discussion

Understanding how viruses respond to ionizing radiation under different physicochemical environments is critical for predicting their persistence in extreme conditions, including those relevant to astrobiology. In this study, we show that the response of bacteriophage Qβ to ionizing radiation is not governed by a single factor, Instead, viral survival is shaped by multiple environmental and viral determinants, with hydration state emerging as the factor with strongest effect among those examined, while mineral matrices and intrinsic viral properties also contribute to the observed patterns of resistance.

### 4.1 Viral concentration influences the apparent dose-response relationship

Our results suggest that the response of bacteriophage Qβ to ionizing radiation may be influenced by the initial concentration of the virus-containing suspension. When dose-response relationships were analyzed using log-transformed viral titers, increasing the initial viral concentration produced a significant displacement of the fitted curves toward higher radiation doses, while no significant differences in slope were detected. This pattern indicates that, under this analytical framework, higher viral concentrations were associated with an apparent shift in the response to radiation without detectable changes in the overall shape of the inactivation curves. However, additional analyses using normalized survival data revealed that the magnitude of this effect depends on the analytical approach employed. Whereas analyses performed within the same log-logistic framework yielded results comparable to those obtained with absolute viral titers, analyses of normalized survival in linear scale did not detect significant differences between concentrations. Therefore, the biological significance of the observed shift should be interpreted cautiously.

In addition, since virus suspensions were obtained by dilution of the same stock preparation, higher viral concentrations were also associated with higher concentrations of co-diluted proteins and other lysate-derived components. Therefore, the present experiments do not allow the observed effect to be attributed exclusively to viral particles.

### 4.2 Mineral matrices modulate radiation response in liquid medium in a composition-dependent manner

The interaction between viral particles and extraterrestrial regolith simulants revealed markedly different effects depending on mineral composition and concentration. The Martian regolith simulant MGS-1, when assayed at a concentration of 12.5% (w/v), modified the response against ionizing radiation in liquid medium, reducing the extent of radiation-induced viral inactivation despite concurrently decreasing basal infectivity. Consistent with this, dose–response analyses showed a rightward shift of the curves together with changes in slope, indicating that MGS-1 affects both the radiation dose required for inactivation and the kinetics of viral decay.

Importantly, the effect of MGS-1 was strongly dependent on its concentration. At a fixed radiation dose of 750 Gy, survival rates across low to intermediate concentrations were similar to, or slightly lower than, those observed in the absence of MGS-1, whereas an increase in survival was observed at the highest concentration tested (100% w/v). Because this experiment evaluated only a single radiation dose, it provides only a partial view of the interaction between regolith concentration and radiation exposure. A more complete characterization would require full dose–response curves across the range of regolith concentrations tested. Nevertheless, the increase in survival observed at 100% (w/v) MGS-1 suggests that additional processes may become relevant at very high regolith simulant concentrations. Under these conditions, the amount of free liquid is substantially reduced, which may limit indirect radiation damage mediated by water radiolysis products. This possibility is consistent with the strong influence of hydration state on viral survival observed elsewhere in this study. However, because only a single radiation dose was evaluated and water availability was not quantified, the potential effect of this mechanism remains unresolved.

Besides the medium-mediated effects described above, direct interactions between viral particles and mineral surfaces may also contribute to the observed protection. Previous studies have shown that MGS-1 can protect microorganisms from polychromatic UV radiation (200–400 nm) at relatively low regolith-to-cell ratios (Zaccaria et al., 2024; Beblo-Vranesevic et al., 2022), and adsorption of viruses onto mineral particles is known to reduce their susceptibility to inactivation and promote persistence in soil environments (Lipson and Stotzky, 1985; Syngouna and Chrysikopoulos, 2010). Assuming that the interaction between MGS-1 and bacteriophage Qβ occurs via adsorption (Arribas Tiemblo et al., 2026b), such interactions could locally shield viral structures or alter their physicochemical environment, thereby contributing to increased resistance. In addition, because dosimetry was not performed within regolith-containing samples, attenuation of the effective radiation dose by the mineral matrix cannot be excluded. Consequently, the effects observed in the presence of MGS-1, both in the dose-response experiment (Figure 2) and at the highest concentration tested in the concentration-response experiment (Figure 3), may result from a combination of adsorption-mediated processes, changes in the local chemical environment, reduced water availability (in the case of the highest MGS-1 concentration), and physical attenuation of the incident radiation.

In contrast, the lunar regolith simulant LMS-1 neither reduced viral infectivity nor showed evidence of a protective effect under the experimental conditions tested (a fixed radiation dose of 750 Gy and different LMS-1 concentrations). A plausible explanation for this difference is that the distinct mineralogical composition and surface physicochemical properties of MGS-1 promote stronger adsorption of viral particles than LMS-1. MGS-1 incorporates a heterogeneous mixture of primary basaltic minerals together with secondary Fe-bearing phases, sulfates and hydrated silica, whereas LMS-1 is composed predominantly of basaltic silicates representative of lunar mare regolith (Cannon et al., 2019; Long-Fox et al., 2023). These compositional differences, together with variations in particle size distribution and surface texture between the simulants, may influence the availability of reactive mineral surfaces for virus adsorption (Long-Fox and Britt, 2023). If virus–regolith interactions are indeed mediated by adsorption, as previously proposed for Qβ in MGS-1 (Arribas Tiemblo et al., 2026b), the greater diversity of adsorption sites in the Martian simulant could stabilize virus–mineral interactions and thereby account for the contrasting radioprotective effects observed in the two simulants.

### 4.3 Hydration state governs viral radiotolerance

One of the most striking findings of this study is that prior desiccation greatly increases the radiation survival of bacteriophage Qβ, regardless of the desiccation methodology employed. Desiccated Qβ populations achieved radiotolerance, defined as the ability to survive exposure to ionizing radiation doses of 3 kGy (Beblo-Vranesevic et al., 2018). This dramatic increase in radioresistance is likely attributable to the removal of water, which reduces the formation and mobility of ROS during irradiation (Mattimore and Battista, 1996). In the absence of water, indirect radiation damage mediated by water radiolysis products—particularly hydroxyl radicals—is minimized, leaving direct radiation damage to nucleic acids and proteins as the primary inactivation mechanism. Similar phenomena have been observed in other microorganisms, including *Deinococcus radiodurans, Escherichia coli*, and *Saccharomyces cerevisiae*, where prior desiccation significantly increased survival rates under gamma radiation (Horne et al., 2022). The consistency of this effect across different desiccation methods further indicates that it is a robust and intrinsic consequence of the dehydrated state, rather than an artifact of a specific experimental procedure. Although both desiccation procedures produced comparable increases in radiation tolerance, the two methods differed in drying time, substrate, and drying environment. Because residual moisture was not directly measured, we cannot determine whether both protocols produced equivalent levels of dehydration. Nevertheless, the consistent increase in survival observed in both approaches supports the conclusion that water availability is a major determinant of radiation sensitivity in bacteriophage Qβ.

An additional factor that may contribute to viral survival under desiccation is the presence of gelatin in the phage buffer. Previous work with bacteriophage Qβ has shown that gelatin and other protein stabilizers such as BSA can substantially reduce desiccation-induced losses of infectivity, suggesting that proteinaceous matrices can protect viral particles during drying (Arribas Tiemblo et al., 2026b). Therefore, the relative contributions of reduced water availability and protein-mediated stabilization cannot be distinguished in the present study. Nevertheless, the marked and reproducible increase in survival observed under two independent desiccation protocols remains consistent with a major influence of hydration state on radiation sensitivity.

### 4.4 A single mutation reveals intrinsic determinants of viral resistance to ionizing radiation

Beyond environmental factors, our results demonstrate that intrinsic properties of the viral particle also play a critical role in determining resistance to ionizing radiation. The A1956G mutation, previously shown to increase resistance to elevated extracellular temperatures (Lázaro et al., 2018), was associated with substantially higher survival following irradiation compared with the wild-type virus. This mutation results in a lysine-to-glutamic acid substitution in the A1 protein, a minor structural component essential for viral infectivity (Hofstetter et al., 1974). Although the precise mechanism remains unknown, the A1956G mutation clearly increases the ability of infectious particles to remain viable following radiation exposure. One possibility is that the K204E substitution enhances the stability of virion structures involved in infectivity. However, because survival was assessed exclusively through plaque assays, the present data do not distinguish between increased structural stability of viral particles and other effects influencing infectivity, such as altered susceptibility of A1 to radiation-induced damage or changes in the relationship between physical particles and infectious units.

An intriguing possibility is that the A1 protein represents a critical target for multiple environmental stressors. This hypothesis is supported by the repeated identification of adaptive mutations in A1 during experimental evolution of Qβ under diverse selective pressures, including low host density, extracellular heat stress, freezing and thawing cycles and simulated microgravity (Lázaro et al., 2018; Laguna-Castro and Lázaro, 2022; Laguna-Castro et al., 2024; Rodríguez-Moreno et al., 2025). Since A1 is necessary for infection, damage affecting this protein could be sufficient to inactivate the virion even if the remainder of the particle remains largely intact. In this context, mutations that reduce the susceptibility of A1 to environmental damage may enhance viral viability under stress conditions, providing a mechanistic basis for the large phenotypic effects observed with small genetic changes. Such a model is consistent with the idea that viral inactivation may be governed by damage to specific vulnerable components rather than by global particle disruption. Although this interpretation is consistent with the observed phenotype, direct evidence identifying A1 as the primary target of radiation-induced inactivation is not currently available.

The cross-protection conferred by the A1956G mutation is consistent with an emerging paradigm in stress biology: resistance mechanisms to desiccation, radiation, and heat may have co-evolved due to their shared reliance on oxidative damage pathways (Fredrickson et al., 2008; Slade and Radman, 2011). In natural arid environments such as deserts, organisms are simultaneously exposed to extreme dryness, intense solar UV radiation, and high daytime temperatures. The overlapping biochemical damage profiles of these stresses—dominated by oxidative modifications to proteins and nucleic acids—may have selected for pleiotropic resistance mechanisms capable of mitigating multiple stress types (Bauermeister et al., 2011). Our findings support this hypothesis and suggest that viral adaptation to one extreme stress can confer latent resistance to others through shared molecular protection strategies.

### 4.5 Implications for viral persistence in extreme environments

Taken together, these observations suggest that the persistence of viral particles in extreme environments cannot be predicted from individual stressors considered in isolation. Instead, survival emerges from interactions between environmental conditions and intrinsic viral properties, with hydration state playing a particularly important role in determining the balance between direct and indirect radiation damage.

These findings have important implications for understanding viral persistence in extreme and extraterrestrial environments. On planetary surfaces such as Mars, where conditions combine desiccation, radiation, and mineral-rich substrates, the fate of viral particles will likely depend on the balance between these interacting factors. In particular, the strong radioprotective effect of desiccation suggests that viruses embedded in dry environments may persist longer than previously anticipated. However, it is important to note that the present experiments were not designed to reproduce complete Martian or lunar environmental conditions. Instead, they address specific factors that may influence viral persistence. Consequently, the results should be interpreted as mechanistic insights into the contribution of these individual factors rather than as direct predictions of viral survival under planetary surface conditions.

## 5 Conclusions

This study demonstrates that the survival of bacteriophage Qβ under ionizing radiation is influenced by multiple environmental and viral determinants. Among the factors examined, hydration state produced the strongest effect, with desiccated samples exhibiting substantially higher survival than hydrated ones. In liquid medium, the Martian regolith simulant MGS-1 modified the response of Qβ to ionizing radiation, whereas LMS-1 showed no evidence of a comparable effect under the experimental conditions tested. In addition, a thermotolerance-associated mutation (A1956G) substantially enhanced viral survival following irradiation, highlighting the importance of viral genetic factors in shaping resistance to environmental stress.

The present study also has several limitations. Mineral-matrix effects and desiccation were evaluated in separate experimental settings, preventing direct assessment of their interactions. Residual moisture content was not quantified in desiccated samples, and dosimetry was not performed within regolith-containing suspensions, making it impossible to determine the relative contributions of physical attenuation, adsorption-mediated processes, and changes in the local chemical environment to the observed protective effects.

Despite these limitations, our results provide an integrated view of how environmental conditions and intrinsic viral properties contribute to the persistence of viruses under radiation stress. Future studies combining mineral matrices, desiccation, and detailed dosimetric analyses may help clarify the mechanisms underlying viral survival in dry, mineral-rich environments relevant to astrobiology and planetary protection.

## Data Availability

The raw data supporting the conclusions of this article will be made available by the authors, without undue reservation.
